# Supplementary material for: Iterative improvement in the automatic modular design of robot swarms
Source: PeerJ Comput Sci. 2020 Dec 7;6:e322. doi: 10.7717/peerj-cs.322 (PMC7924708; doi:10.7717/peerj-cs.322)
Supplement: Supplemental Information 3 [file peerj-cs-06-322-s003.zip › argos3/doc/api/standalone/a00517_source.html]

ARGoS: plugins/robots/generic/control\_interface/ci\_range\_and\_bearing\_sensor.h Source File


- Main Page
- Related Pages
- Namespaces
- Classes
- Files

- File List
- File Members

# plugins/robots/generic/control\_interface/ci\_range\_and\_bearing\_sensor.h

Go to the documentation of this file.

```
00001 
00007 #ifndef CI_RANGE_AND_BEARING_SENSOR_H
00008 #define CI_RANGE_AND_BEARING_SENSOR_H
00009 
00010 namespace argos {
00011    class CCI_RangeAndBearingSensor;
00012 }
00013 
00014 #include <argos3/core/control_interface/ci_sensor.h>
00015 #include <argos3/core/utility/datatypes/datatypes.h>
00016 #include <argos3/core/utility/datatypes/byte_array.h>
00017 #include <argos3/core/utility/math/quaternion.h>
00018 
00019 namespace argos {
00020 
00021    /****************************************/
00022    /****************************************/
00023 
00024    class CCI_RangeAndBearingSensor : public CCI_Sensor {
00025 
00026    public:
00027 
00028       struct SPacket {
00029          Real Range;
00030          CRadians HorizontalBearing;
00038          CRadians VerticalBearing;
00039          CByteArray Data;
00040 
00041          SPacket();
00042       };
00043 
00044       typedef std::vector<SPacket> TReadings;
00045 
00046    public:
00047 
00048       virtual ~CCI_RangeAndBearingSensor() {}
00049 
00050       const TReadings& GetReadings() const;
00051 
00052 #ifdef ARGOS_WITH_LUA
00053       virtual void CreateLuaState(lua_State* pt_lua_state);
00054 
00055       virtual void ReadingsToLuaState(lua_State* pt_lua_state);
00056 #endif
00057 
00058    protected:
00059 
00060       TReadings m_tReadings;
00061 
00062    };
00063 
00064    /****************************************/
00065    /****************************************/
00066 
00067 }
00068 
00069 #endif
```

---

Generated on 10 Jul 2018 for ARGoS by 
 1.6.1
